# Editorial: Oral health care for vulnerable and underserved populations

**DOI:** 10.3389/froh.2025.1633512

**Published:** 2025-08-06

**Authors:** Adriana Modesto, Apoena Aguiar Ribeiro

**Affiliations:** ^1^ Department of Pediatric Dentistry, School of Dental Medicine, University of Pittsburgh, Pittsburgh, PA, United States; ^2^ Department of Diagnostic Sciences, Adams School of Dentistry, University of North Carolina, Chapel Hill, NC, United States

**Keywords:** oral health, disparities, vulnerable, equity, oral healthcare access

**Editorial on the Research Topic**
Oral health care for vulnerable and underserved populations

In the shadow of modern dentistry's technological achievements lies a troubling reality: millions of vulnerable individuals face significant barriers to basic oral healthcare, which in turns have a direct effect on their quality of life. These disparities represent not just a clinical challenge but a social justice issue demanding our collective attention.

According to the Department of Health and Human Services, “underserved communities” include populations sharing characteristics that have been systematically denied full participation in economic, social, and civic life. These vulnerable groups—racial and ethnic minorities, children, elderly individuals, the socioeconomically disadvantaged, the underinsured, and those with certain medical conditions—often bear a disproportionate burden of oral disease with far-reaching consequences for their overall health.

This Special Issue brings recent research that illuminates both these disparities and promising pathways toward more equitable oral healthcare systems. These studies document challenges faced by diverse vulnerable populations while highlighting innovative approaches that could transform oral healthcare delivery.

## Diverse populations, shared challenges

Among indigenous communities, researchers examining the Irula tribes of Tamil Nadu, India, are conducting a cross-sectional study of 880 individuals aged 60 and above to quantify tooth morbidity while identifying risk factors and assessing oral hygiene practices. The study highlights how geographic isolation compounds vulnerability, with remote locations severely limiting access to fundamental healthcare services Sukumar et al.

For socially marginalized youth, a comprehensive review spanning four countries identified three major obstacle categories: financial constraints, structural challenges, and psychological barriers. These impediments contribute to alarming rates of unmet dental needs. Disturbingly, after screening 484 studies, researchers found only seven relevant publications, underscoring a critical research gap Vaishampayan et al. Even populations with presumed advantages face challenges. International post-secondary students consistently experience poorer oral health outcomes compared to domestic peers—exhibiting knowledge gaps, less favorable hygiene behaviors, and lower utilization of routine dental care. This vulnerability stems from multiple factors including acculturation stress, financial constraints, and dietary changes Yassin et al.

Research examining oral health in opioid-addicted patients reveals profound disparities. The University of Regensburg study documented significantly poorer oral health indicators—including dramatically higher DMFT scores (median 21 vs. 10 in controls), greater periodontal treatment needs, and lower salivary pH levels among opioid users. These findings highlight the complex interrelationship between substance use disorders and oral health.

For elderly populations, analysis of odontogenic abscesses reveals important distinctions. A seven-year study of 1,173 hospitalized patients found that elderly patients experienced longer hospital stays and higher complication rates. Chronic renal failure emerged as a significant predictor of adverse outcomes, pointing to the need for specialized approaches for geriatric dental patients Kaercher et al.

## From clinical advances to structural reforms

Recent innovations offer promises for extending quality oral healthcare to vulnerable populations. A transformative study demonstrates success treating trauma-induced tooth loss using a novel 4-axial implant-based protocol. This approach achieved 100% implant retention over 48 months with minimal bone loss, potentially revolutionizing treatment for patients historically lacking access to sophisticated restorative options Wang et al.

Beyond clinical innovations, structural approaches show promise for expanding access. Hungary's implementation of dental clusters offers insights into reorganizing primary dental care. Financial incentives and professional development opportunities motivated practitioners to join these clusters, now encompassing one-third of dental practices since 2021 Sztrilich et al.

While barriers exist, including information gaps and practitioner distrust, analysis suggests considerable potential for service expansion and digital health integration. Successful widespread adoption requires thoughtful policy measures addressing practitioners' implementation concerns.

## Bridging research, policy, and practice

These studies collectively illuminate several priorities for addressing oral health disparities:

First, we must expand research to address critical knowledge gaps. The lack of robust studies on marginalized populations underscores the need for more diverse investigations employing rigorous methodologies while centering perspectives of affected communities (1, 2).
Second, interventions must be tailored to specific vulnerable populations. Whether addressing elderly patients with complex comorbidities, individuals with substance use disorders, indigenous populations, or international students navigating unfamiliar healthcare systems, interventions must be culturally appropriate and target specific barriers (1–4).
Third, structural reforms in healthcare delivery models hold promise for expanding access. However, successful implementation requires addressing both provider concerns and patient needs through thoughtful policy design (5).

Most importantly, meaningful progress requires community engagement. Effective program design necessitates participatory research prioritizing understanding of vulnerable populations’ unique perspectives and needs (3).

As dental professionals, researchers, policymakers, and advocates, we share a collective responsibility to address these persistent inequities. The research highlighted here offers both sobering evidence of challenges and inspiring examples of solutions. By combining clinical innovation with structural reform and community engagement, we can build oral healthcare systems serving all populations—particularly those most vulnerable.

**Figure 1:**
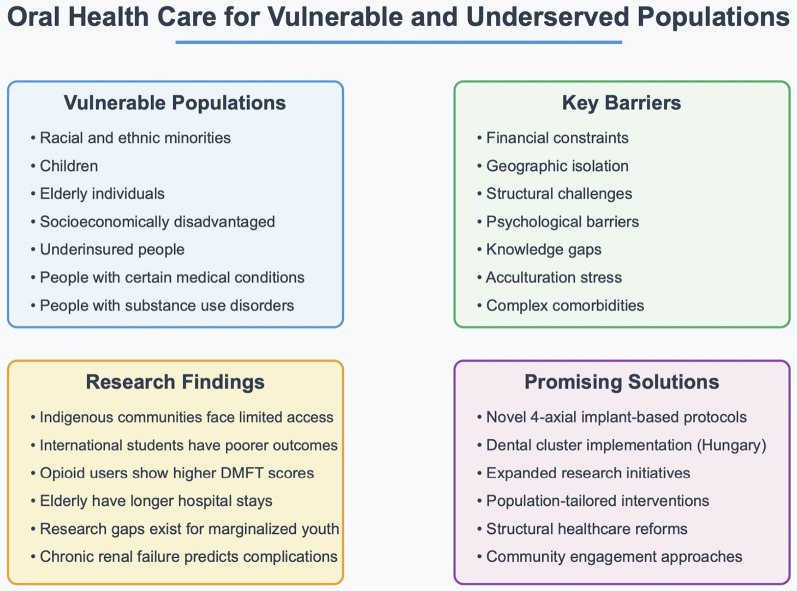
Key barriers to promote oral health to vulnerable populations, and research findings that can bring innovative solutions to improve knowledge and access to oral health.

In conclusion, the scientific literature has a growing consensus that researchers, including health professionals and policymakers, must expand oral health research to address critical knowledge gaps affecting marginalized populations (Figure 1). Multiple reviews highlight the lack of robust, methodologically diverse studies focusing on socially and structurally disadvantaged groups, including Indigenous communities, migrants, elderly, and individuals facing severe and multiple disadvantages. These gaps limit our understanding of the unique barriers these populations face and hinder the development of effective, equitable interventions. To advance oral health equity, it must prioritize inclusive designs, involve larger and more representative samples, and adopt community-centered approaches that elevate the voices and lived experiences of those most affected. Embracing rigorous methodologies and culturally responsive frameworks is essential to produce evidence that informs meaningful, systemic change. Achieving oral health equity is not merely a technical challenge but a moral imperative. Access to quality oral healthcare is a fundamental component of human dignity that cannot remain a privilege for some but must become a reality for all.

## References

[1] Verma A Priyank H Renuka P Kumari M Sayed Abdul N Shivakumar S. A systematic review and meta-analysis on oral health disparities among the indigenous paediatric population. Cureus. (2023) 15(7):e41673. 10.7759/cureus.41673 37575701 PMC10412898

[2] Feitosa J Hagenbuch S Patel B Davis A. Performing in diverse settings: a diversity, equity, and inclusion approach to culture. Int J Cross Cult Manag. (2022) 22(3):433–57. 10.1177/14705958221136707

[3] Ponce-Gonzalez I Cheadle A Aisenberg G Cantrell LF. Improving oral health in migrant and underserved populations: evaluation of an interactive, community-based oral health education program in Washington State. BMC Oral Health. (2019) 19(1):30. 10.1186/s12903-019-0723-7 30760255 PMC6375135

[4] Tsai C Blinkhorn A Irving M. Oral health programmes in indigenous communities worldwide-lessons learned from the field: a qualitative systematic review. Community Dent Oral Epidemiol. (2017) 45(5):389–97. 10.1111/cdoe.12302 28425612

[5] Kruk ME Gage AD Arsenault C Jordan K Leslie HH Roder-DeWan S High-quality health systems in the sustainable development goals era: time for a revolution. Lancet Glob Health. (2018) 6(11):e1196–252. 10.1016/S2214-109X(18)30386-3. Erratum in: Lancet Glob Health. (2018) 6(11):e1162. doi: 10.1016/S2214-109X(18)30438-8. Erratum in: Lancet Glob Health. (2018) 6(11):e1162. doi: 10.1016/S2214-109X(18)30456-X. Erratum in: Lancet Glob Health. (2021) 9(8):e1067. doi: 10.1016/S2214-109X(21)00250-3. 30196093 PMC7734391

